# Association of Trauma from Occlusion with Localized Gingival Recession in Mandibular Anterior Teeth

**Published:** 2009

**Authors:** Pratibha Panduranga Kundapur, Khandige Mahalinga Bhat, Giliyar Subraya Bhat

**Affiliations:** * MDS, Associate Professor, Department of Periodontics, Manipal College of Dental Sciences, Manipal, India; ** MDS, Professor, Department of Periodontics, Manipal College of Dental Sciences, Manipal, India; *** MDS, MFGDP, Professor and Head, Department of Periodontics, Manipal College of Dental Sciences, Manipal, India

**Keywords:** Dental occlusion, Gingival recession, Incisor, Tooth mobility, Traumatic

## Abstract

**Background:**

There have been passing references in history that excessive occlusal forces might be a causative factor in gingival recession. The purpose of the present cross-sectional study was to explore the role of trauma from occlusion on the development of gingival recession.

**Methods:**

Three hundred patients reporting to the department of Periodontics were screened for the presence of gingival recession in the lower incisors. A single trained examiner carried out clinical examination for signs of trauma from occlusion, such as fremitus test, presence of wear facets and mobility. The data were analyzed by chi square test.

**Results:**

No statistically significant relationship was observed between the presence of a positive fremitus and wear facets with gingival recession. However, a significant association was observed between patients who experienced mobility and gingival recession.

**Conclusion:**

There does appear to be a relationship between fremitus and tooth wear with gingival recession based on the results of the present study, though not conclusive. However, the sign of tooth mobility, which is a feature of trauma from occlusion, appeared to be a predictor of positive association with gingival recession.

## Introduction

Occlusal forces were, at one time, implicated in the progression of periodontal disease.^1^ However, there is very little conclusive evidence to support this statement. At present, there is a consensus that trauma from occlusion may be a co–destructive factor in periodontal destruction, especially affecting the supporting alveolar bone.^2^ Nevertheless, opinions are divided on its role in affecting the periodontal attachment, i.e., marginal gingival tissues. The purpose of this cross-sectional study was to explore the role of trauma from occlusion on the development of gingival recession. Gingival recession is the denudation of the tooth cementum by an apical shift in the position of the gingiva in the direction of root apex. Gingival recession in relation to mandibular anterior teeth poses problems of esthetics and increases the likelihood of developing hypersensitivity and root caries. A 70% prevalence of gingival recession has been reported in the literature.^3^ Therefore, a proper understanding about the etiology causes involved directly or indirectly in the causation of gingival recession is important. Historically, it has been suggested that excessive occlusal force might be a causative factor in gingival recession. Stillman was perhaps the first to describe a specific type of gingival recession, a narrow triangular shaped cleft on the facial aspect of the tooth.^4^ Box (1930), Miller (1934) and McCall (1921) advocated the concept that recession was caused by trauma to the periodontium as a result of occlusal interferences.^5^ They had noted that, teeth which exhibited gingival recession commonly had signs and symptoms of traumatic occlusion, such as wear facets, heavy occlusal contacts, etc. There is much disagreement on whether occlusal trauma contributes to or causes gingival recession. Harrel and Nunn^6^ reported that more than 70% of teeth with functional disturbances were associated with gingival recession. Gorman^7^ who evaluated 164 patients with gingival recession, however, could not relate the presence of recession to occlusal trauma. Bernimoulin and Curilovie^8^ evaluated 107 teeth with mobility and gingival recession but found no correlation between the two. Hence, insufficient evidence in this area prompted us to further investigate the possibility of an association between the presence of signs of trauma from occlusion and the development of gingival recession. The present study intended to determine the prevalence of trauma from occlusion in subjects with localized gingival recession in the mandibular anterior teeth with the aim of relating the association of trauma from occlusion as an etiological factor in gingival recession.

## Materials and Methods

Patients in the age group of 14-55 years reporting to the department of Periodontics, Manipal College of Dental Sciences, Manipal, India were screened for the presence of gingival recession from June 2007 to August 2007. They were recruited into the study on a voluntary basis. All participating subjects were required to sign an informed consent form. At the conclusion of the clinical examination, participants were informed about their oral hygiene status and any diagnosed periodontal pathology. Patients with diagnosed pathological conditions were offered appropriate treatment. A total of 300 patients with gingival recession were included in this cross–sectional study. Patients were excluded if they had a history of previous periodontal therapy, occlusal adjustment, missing anterior teeth or orthodontic treatment. Patients with significant systemic disease and pregnant women were also excluded. In order to collect the data, an interview cum dental examination with the help of a proforma prepared for the study was carried out. A single trained examiner performed the periodontal examination of the mandibular anterior region for each of these patients to avoid interexaminer bias.

The following parameters were recorded. Plaque was scored on a range of 0 to 3 using Plaque Index of Silness and Loe.^9^ Detection of supragingival/subgingival calculus was marked as present or absent. Severity of gingivitis was assessed by using Loe and Silness gingival index.^10^ Presence or absence of gingival recession was noted and further recorded according to the classification proposed by Miller Jr.^11^ Presence/absence of wear facets were recorded. The periodontal examination comprised assessments of tooth mobility (Miller’s index) and fremitus test (functional mobility). Tooth mobility was recorded using the Miller’s mobility index.^12^ Presence/absence of fremitus was detected by placing the index finger on the facial surfaces of maxillary anterior teeth during repeated habitual centric closure. An experienced technician took standardized periapical radiographs of the involved teeth only in doubtful cases. Statistical calculations were carried out using SPSS version 10. Standard descriptive statistics were used to summarize the variables studied. Possible association between clinical signs of trauma from occlusion and gingival recession was analyzed using the chi square test.

## Results

Data were collected from 300 patients who had sought consultation at the department of Periodontics; 207 (69%) males and 93 (31%) females with a mean age of 33 ± 9.56 years were enrolled in the study. Out of 300 subjects, 108 (36%) reported for routine check up without any specific complaints. Nine (3%) complained of persistent discomfort upon eating or pain. Fortysix (15.3%) patients complained of sensitivity of teeth and 11 (3.7%) patients noticed drifting of teeth. One (0.3%) patient experienced both persistent discomfort and drifting of teeth. Eleven (3.7%) patients complained of persistent discomfort and sensitivity. Three subjects (1%) reported drifting as well as sensitivity of teeth; 111 (37%) patients had other complaints that did not require periodontal therapy.

Excellent oral hygiene status was observed in 87 (29%) patients while 149 (49.7%) ones had good oral hygiene; 43 (14.3%) patients were observed to have fair oral hygiene and 21 (7%) had poor oral hygiene. Mild gingivitis was observed in 263 (87.7%) patients. Moderate gingivitis was noted in 31 (10.3%) patients. Six patients (2%) had severe gingivitis. Calculus was observed in 197 (65.7%) patients. Out of 300 patients, 159 (53%) were found to have gingival recession. Class I recession was observed in 112 patients (37.3%), class II in 45 (15.0%) patients and class III in 2 (7%) patients. Tables 1, 2 and 3 show the association of fremitus test, tooth wear and tooth mobility with gingival recession respectively. Radiographs were advised in 36 patients to have additional evidence for considering the presence of trauma from occlusion. Widened periodontal ligament space was observed in 26 of these patients (8.7%), both widening of periodontal ligament space and bone loss were observed in 8 patients (2.7%) and widening, bone loss and lamina dura thickening was noticed in 2 patients (0.7%).

**Table 1:** Association of fremitus test with gingival recession.

Fremitus test	Recession		Total
	With recession n (%)	Without recession n (%)	n (%)
Present	94 (59.1)	76 (53.9)	170 (56.7)
Absent	65 (40.9)	65 (46.1)	130 (43.3)
Total	159 (100)	141 (100)	300 (100)

Chi square test: Continuity correction = 0.630, df = 1, P= 0.427

**Table 2:** Association of tooth wear with gingival recession.

Wear facets	Recession		Total
	With recession n (%)	Without recession n (%)	n (%)
Present	143 (89.9)	125 (88.7)	268 (89.3)
Absent	16 (10.1)	16 (11.3)	32 (10.7)
Total	159 (100)	141 (100)	300 (100)

Chi square test: Continuity correction = 0.030, df = 1, P = 0.863

**Table 3:** Association of tooth mobility with gingival recession.

Mobility	Recession		Total
	With recession n (%)	Without recession n (%)	n (%)
PRESENT	55 (34.6)	11 (7.8)	66 (22)
ABSENT	104 (65.4)	130 (92.2)	234 (78)
TOTAL	159 (100)	141 (100)	300 (100)

Chi square test: Continuity correction = 29.713, df = 1, P < 0.001 Odd’s ratio: 6.2; C.I = 3.1 to 12.5 The odds of developing recession among those with mobility is 6.2.

## Discussion

It is generally observed that gingival recession in relation to a tooth is coupled with traumatic occlusal forces. Yet, there is reluctance to relate trauma from occlusion as an etiological factor in gingival recession and is expressed only in a murmur or hushing voice. The condition is more pronounced in the mandibular anterior region, especially in cases of increased overbite. This situation being a common observation during everyday dental practice, prompted us to evaluate the truth by doing a preliminary investigation. The present study was conducted to determine the prevalence of trauma from occlusion in subjects with localized gingival recession in the mandibular anterior teeth. Further, we tried to relate the association of trauma from occlusion as an etiological factor for gingival recession in these teeth. For this purpose, the teeth were clinically examined for the presence of gingival recession. Most patients selected had good oral hygiene with minimal gingival inflammation. To assess the effects of trauma from occlusion, fremitus test, tooth mobility and presence of wear facets were used as criteria. Prevalence of a positive fremitus test in patients with gingival recession was found to be 59.1%.(Table 1) There was no significant association between fremitus test and gingival recession; 59.1% of the individuals with recession exhibited fremitus whereas 53.9% without recession also exhibited fremitus was not detected at the chair side. Abnormal contacts during these parafunctional movements may have gone unnoticed.

Our results were consistent with the findings of Gorman,^7^ who evaluated 164 patients with gingival recession and could not relate the presence of recession to occlusal trauma. However, Harrell and Nunn^6^ observed that more than 70% of teeth with functional disturbances were associated with gingival recession. Jin and Cao^13^ have also reported that teeth with significant fremitus had more attachment loss and less osseous support. No significant association was evident between the presence of wear facets and the development of gingival recession. (Table 2) Among patients with gingival recession, 89.9% had wear facets compared to 88.7% of patients without recession. This may be attributed to an inherent capacity of the periodontium to withstand forces to within a physiologic limit beyond which it would manifest as loss of attachment. These results were inconsistent with those of Jin and Cao^13^ where in it was observed that teeth with pronounced wear had less attachment levels.

A significant association was observed between the presence of tooth mobility and gingival recession. (Table 3) 34.6% of patients with gingival recession had tooth mobility as compared to only 7.8% of patients without gingival recession. The odds of developing recession in patients who have mobility were about 6.2. Wang et al^14^ observed significant attachment loss in teeth with mobility compared to those without mobility. This was in contrast with the findings of Bernimoulin and Curilovie^8^ who on evaluating 107 teeth with mobility and gingival recession found no correlation.

The findings of the present study make one feel that presence of a positive fremitus test need not summarily be conclusive for existence of trauma from occlusion. However, it would not be wrong to surmise that the occurrence of all three factors, namely, fremitus with positive wear facets and mobility, even without radiographs would indicate trauma from occlusion. The present study confines clinical assessment of changes due to trauma from occlusion to the anterior teeth only. A more detailed assessment of the dentition as a whole would give a more accurate reflection of the intended results.

## Conclusion

From this cross-sectional study, it may be concluded that:

1. The prevalence of positive fremitus test in patients with gingival recession was about 59.1%.
2. Fremitus was not significantly associated with the development of gingival recession.
3. Wear facets were not significantly associated with the presence of gingival recession.
4. Mobile teeth were significantly associated with the development of gingival recession.
5. The odds of developing gingival recession in mobile teeth are about 6.2 as compared to non-mobile teeth.
